# GAN-Based Video Denoising with Attention Mechanism for Field-Applicable Pig Detection System

**DOI:** 10.3390/s22103917

**Published:** 2022-05-22

**Authors:** Zhao Bo, Othmane Atif, Jonguk Lee, Daihee Park, Yongwha Chung

**Affiliations:** 1 Department of Computer and Information Science, Korea University, Sejong Campus, Sejong City 30019, Korea; ku_bozhao@korea.ac.kr (Z.B.); osuman@korea.ac.kr (O.A.); 2 Department of Computer Convergence Software, Korea University, Sejong Campus, Sejong City 30019, Korea; ychungy@korea.ac.kr

**Keywords:** pig detection, IR reflection, attention mechanism, GAN, image dehazing

## Abstract

Infrared cameras allow non-invasive and 24 h continuous monitoring. Thus, they are widely used in automatic pig monitoring, which is essential to maintain the profitability and sustainability of intensive pig farms. However, in practice, impurities such as insect secretions continuously pollute camera lenses. This causes problems with IR reflections, which can seriously affect pig detection performance. In this study, we propose a noise-robust, real-time pig detection system that can improve accuracy in pig farms where infrared cameras suffer from the IR reflection problem. The system consists of a data collector to collect infrared images, a preprocessor to transform noisy images into clean images, and a detector to detect pigs. The preprocessor embeds a multi-scale spatial attention module in U-net and generative adversarial network (GAN) models, enabling the model to pay more attention to the noisy area. The GAN model was trained on paired sets of clean data and data with simulated noise. It can operate in a real-time and end-to-end manner. Experimental results show that the proposed preprocessor was able to significantly improve the average precision of pig detection from 0.766 to 0.906, with an additional execution time of only 4.8 ms on a PC environment.

## 1. Introduction

Pork is the most consumed meat product worldwide. Over 100 million metric tons of pork are produced and consumed every year [1], primarily from intensive pig farms. The profitability and sustainability of intensive pig farms are affected by problems relating to pigs’ health and welfare [2]. The successful management of pig health and welfare problems requires lengthy and accurate human observation, which can be challenging for commercial pig farms for the following reasons: (1) more than 1000 pigs are typically managed by each staff member in most intensive pig farms, which makes routine daily checks on the health situation of each pig impractical [3]. (2) Observing each pig for a short period is very likely to overlook subtle changes in behavior, which can help assess pigs’ health and diagnose some diseases at an early stage [4]. (3) Judgments made by human experts tends to be affected by bias on the part of observers, which impacts the accuracy and reliability of the results [5]. Automatic pig monitoring systems have recently become popular alternatives to human observation. They provide consistent, objective, and accurate observations of pig health and welfare with limited human intervention.

Recently, many studies have been conducted on automatic livestock monitoring using various methods. Some of these include wearable sensor-based [6,7,8], audio-based [9,10,11,12,13], or video-based methods [14,15,16,17,18,19,20,21,22]. Compared with sensor-based methods, video-based methods can provide a non-invasive, non-stressful, and intuitive way to monitor the behavior of an individual or a group of pigs where the sensor-based method fail to do. Compared with audio-based methods, video-based methods can intuitively grasp a pig’s health condition. In contrast, specialists are always required for further sound analysis and recognition in audio-based methods [9,10,11]. Thus, video-based methods have received the most attention in recent studies. Although different types of cameras, such as depth, RGB, and infrared cameras, have been used in video-based methods, infrared cameras are the most widely used in recent research for 24 h continuous pig monitoring systems. They are preferred because they are the least affected by changes in illumination, especially because they can deliver useful image data even at night when most farms turn off their artificial lights [14,15,16,17].

**Figure 1:**
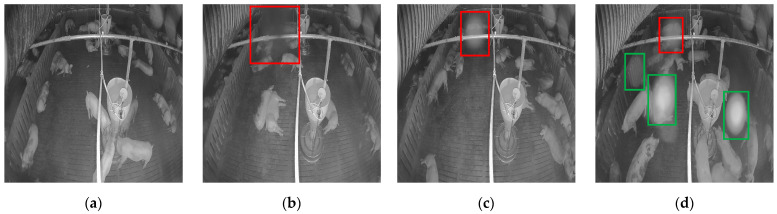
The formation of a haze spot caused by insect secretion: (**a**) clean image before the appearance of a haze spot; (**b**) an insect lands on the camera; (**c**) a haze spot formed after the insect left; (**d**) more haze spots appeared a few days later.

Although infrared cameras are suitable for automatic pig monitoring for the reasons mentioned above, in practice, the frames received from the camera tend to include noise that can severely affect pig detection performance, which is the first process for further pig behavior analysis. This practical problem is caused by the harsh environment of typical pig farms, in which a large number of insects flying around the pigpen often deposit secretions on the cameras. When infrared cameras are recording, they emit infrared rays to capture images. Thus, insect secretions attached to the camera reflect the infrared light directly, causing an IR reflection problem and forming a haze spot phenomenon. Figure 1 shows the process of haze-spot formation caused by insect secretions. Figure 1a shows a clean image captured after the camera installation. However, one week later, an insect appeared on the camera (see the red box in Figure 1b) and formed a haze spot (see the red box in Figure 1c), and a few days later, more haze spots were formed on the frame (see the green boxes in Figure 1d). The cameras in pig farms are typically only cleaned at regular intervals. Thus, owing to the frequent occurrence of such noise, physically cleaning each camera when a haze spot appears is practically impossible. The emergence of these haze spots has a serious impact on the detection of individual pigs. For example, pigs covered by haze spots are difficult to detect using object detection models because of occlusion. In this context, it is necessary to remove the haze spots in noisy images to obtain corresponding clean images as a preprocessing step to maintain the performance of the automatic pig detection modules generally used in pig monitoring systems.

The haze spot in the picture above has a similar property in terms of color and transmission as the haze that appears in image dehazing problems. The image dehazing task aims at recovering haze-free images from images containing haze caused by atmospheric absorption and scattering light, and many breakthrough studies have been reported recently in this field of research [23,24,25,26,27,28,29,30,31,32,33]. However, although image dehazing problems and the mentioned haze spot removal problem introduced earlier share a lot in common, the haze spot removal problem is more challenging. Some reasons for this are given as follows: (1) The color of haze spots is very similar to the color of pigs and obstacles near the camera, such as the pipe in the middle of the image. (2) Haze spots can appear anywhere in an image, and the number and intensity of haze spots are highly unpredictable. (3) The profile of the haze spot is apparent compared with the haze in the image dehazing problem, which can cover the entire image. Therefore, models constructed for image dehazing may not concentrate sufficiently on specific haze spot areas in haze-spot removal problems. (4) Owing to the movement of pigs, it is impossible to acquire the paired data of clean images and images with haze spots required to train an accurate model to transform a noisy image into a clean image. For these reasons, the models used to address the image dehazing problem are not suitable to solve the haze spot removal problem.

In this study, we propose a noise-robust, real-time, infrared camera-based pig detection system that can be used for 24 h monitoring of real pig farms affected by haze spots. The system first uses an end-to-end point attention-guide preprocessor to transform a noisy image into a clean image. The preprocessor embeds a multi-scale learnable attention module into a U-Net network [34] and a generative adversarial network (GAN) [35]. It first locates haze spots in noisy images at a multi-feature level and then obtains an accurate corresponding clean image. Finally, a detector is used to detect pigs based on YOLOv5. Among the most recently released detectors, this model exhibits the advantages of rapid inference speed, high detection accuracy, and a lightweight network [36,37].

The key contribution of this paper is the proposed efficient image denoising model that we built by embedding several trainable attention modules into a U-Net network. The attention modules were introduced to the network because of their effectiveness in improving a model’s performance by enabling it to pay more attention to the region of interest [38]. By adding them to our model, these attention modules help the denoising network master the position and degree of the haze spot in an image. As a result, the denoising model is able to strengthen the image transformation in the area of the noise between the noisy and clean image while suppressing the transformation in the clean area, guaranteeing by that a better denoising performance. Our paper’s contributions are summarized as follows:

1. A multi-scale attention-guided preprocessor is proposed to focus the model on haze spot areas at a multi-scale feature level and obtain an accurate, clean image.
2. A noise simulation method is proposed to simulate haze spots, and the preprocessor was trained on paired noisy and clean data.
3. A multiscale attention loss function is proposed to accelerate the attention module in the preprocessor regression to the attention map.
4. The proposed method significantly improved the performance of pig detection in actual pig farms subject to frequent camera pollution, and outperformed state-of-the-art techniques.

## 2. Related Work

**Figure 2:**
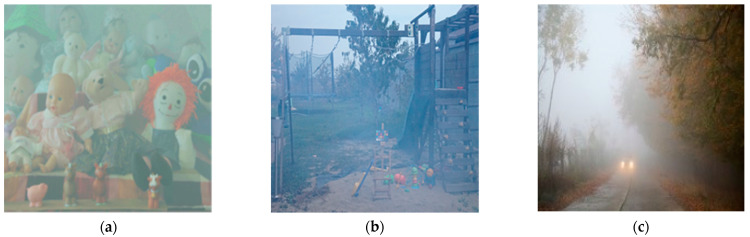
Examples of images with haze: (**a**) simulated indoor haze image from I-Haze dataset [39]; (**b**) simulated outdoor haze image from O-Haze dataset [40]; (**c**) real haze image.

The haze spot removal problem is similar to the image dehazing problem, which aims to restore a clean image from a hazy image caused by fog, haze, or smoke. Figure 2 shows examples of hazy images.

The formation of haze in images can be described by an atmospheric scattering model

(1) I(x)=J(x)t(x)+A(1−t(x))

where I(x) and J(x) represent the hazy and clean images, respectively. t(x) is the transmission map and A is the global atmospheric light. When atmospheric light is homogeneous, $t\left(x\right)=e^{-\beta d\left(x\right)}$ where β refers to the attenuation coefficient, and d(x) refers to the depth information of the scene.

Recently, many studies have been conducted to address the image-dehazing problem. These methods may be generally divided into two approaches, including intermediate-based methods and end-to-end based methods.

Intermediate-based methods first estimate a transmission map t(x) and an atmospheric light A, and then restore a clean image by applying the atmospheric scattering model. Intermediate-based methods can be classified as prior-based methods [23,24] and learning-based methods [25,26]. The prior-based method estimates a transmission map by hand-crafted priors that are designed to distinguish between hazy and clean images, such as the dark channel prior [23] and color attenuation prior [24]. The learning-based methods estimate the intermediate parameters by learning a transformation from the haze image to the transmission map t(x) and atmospheric light A using convolutional neural networks (CNNs), such as DehazeNet [25] and multi-scale CNN [26]. The intermediate-based method is not applicable to our haze spot-removing problem because it is impossible to acquire the transmission map t(x) and atmospheric light A from the haze spot image (Figure 1c).

The end-to-end based method, which is the most recent trend for the image dehazing problem, learns a pixel-to-pixel mapping between hazy and clean images. Thus, this method can directly transform a hazy image into a clean image without using the atmospheric scattering model. An end-to-end deep-dehaze network was proposed in [30] by embedding a residual network into U-Net, which has a symmetrical structure comprising a contracting encoder, an expanding decoder, and a skip connection between the encoder and decoder that can retrieve the missing spatial information. GridDehazeNet [31] integrated an attention mechanism, which encouraged the model to enhance the vital features of the input image into a grid network. The results demonstrated the efficiency of the model. More recently, to make the transformed haze-free image as similar to the clean image as possible, some studies [27,28,29] have constructed a dehazing model by utilizing GANs. This approach can generate more realistic images and has exhibited promising performance in various types of image-to-image translation tasks, such as image super-resolution [41] and image segmentation [42]. GANs consist of two sub-models, including a generator trained to generate new samples and a discriminator trained to judge whether a sample is real or generated. These two sub-models are trained in a competitive manner, in which the generator learns to generate a sufficiently realistic sample such that the discriminator cannot distinguish them from real samples. An end-to-end image dehazing model based on a conditional GAN [42] was proposed in [29] as the conditional GAN provides conditional information that can increase the stability during the training process and improve the representation ability of the generator compared to previous models. Owing to the lack of paired real haze and haze-free images, the Cycle-Dehaze network [28] was introduced using CycleGAN [43], which can be trained by unpaired haze and clean images. Subsequently, an Enhanced Pix2pix Dehazing Network was proposed [27] to generate more realistic haze-free images. This network added an enhanced network following the generator, to reinforce the output image. The end-to-end-based method is suitable for our target application because it can transform noisy images into clean images directly without calculating intermediate parameters, as is the case in the intermediate-based method.

Table 1 summarizes some recent studies on the image dehazing problem. We compared the haze-spot-removing problem with the image-dehazing problem. Although they share many similarities in terms of color properties and transmission, there are still many differences between them. For example, the haze in the dehazing problem is successive, appears in the entire image, and its intensity is related to the depth of the scene. In contrast, the haze spots in the problem described above are discretely distributed, and their intensities and positions are random and unpredictable. Although methods previously proposed to solve the image dehazing problem can remove haze spots to some extent, they cannot obtain promising results because they consider each area in the noisy image equally. Hence, a spatial attention-based method is necessary to force the denoising model to concentrate on the haze spot area. Thus, in this study, we propose a denoising model that embeds a multi-feature level spatial attention module into U-net and GAN models. The model can grasp the intensity and position of the haze spot and strengthen the transformation between noisy and clean images in the haze spot region while suppressing the transformation in the clean region. To the best of our knowledge, the present work is the first to consider the removal of this particular type of noise from images using a deep-learning method.

## 3. Noise-Robust Real-Time Infrared Camera-Based Pig Detection System

**Figure 3:**
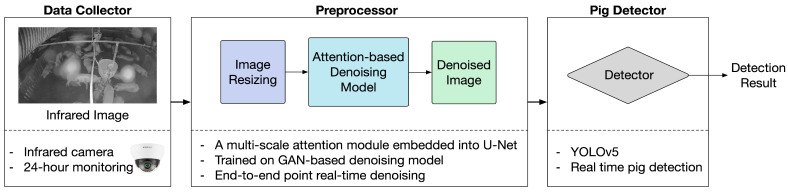
Overall structure of the proposed noise-robust, real-time, camera-based pig detection system.

The structure of the proposed noise-robust, real-time, camera-based pig detection system is illustrated in Figure 3. It mainly consists of three parts: a data collector, a preprocessor, and a pig detector.

### 3.1. Data Collector and Preprocessor

Image data are recorded using an infrared dome camera installed in a pigpen and transmitted to a preprocessor. In the preprocessor, the noisy image is first transformed from a single-channel grayscale image into a three-channel image by duplicating the single-channel data into two more channels because the following denoising and object detection models were originally designed for three-channel RGB image transformation [42] and object detection [37]. Then, the image is resized to a defined size and transformed into the corresponding clean image using a real-time, end-to-end point, attention-based denoising model.

The denoising model is trained based on a GAN approach which consists of a generator and a discriminator. During the training, first, the generator is trained to learn a transformation from the input noisy image to the output clean image. Then, the clean images generated by the generator are sent to the discriminator which is continuously being trained to improve its ability to distinguish the generated clean images from the real clean images. Every time the discriminator outputs a result of its distinction between real and generated images, this result gets fed back to the generator which is then trained to generate a better clean image that may fool the discriminator. By training the generator and discriminator alternately, the generator can generate a sufficiently clean image from a noisy image it receives as input. The generator used in this paper is an attention-based U-Net network that can produce a cleaner image by locating the specific noisy regions in a noisy image fed in a multi-scale feature level through several attention modules that can be trained in a supervised way. As for the discriminator, it is derived from conditional GAN [42] and has the advantage of providing a more stable and faster training by incorporating additional information. The following sub-section introduces in more detail the architecture of the generator and the discriminator, and the loss function used for training.

#### 3.1.1. Generator

**Figure 4:**
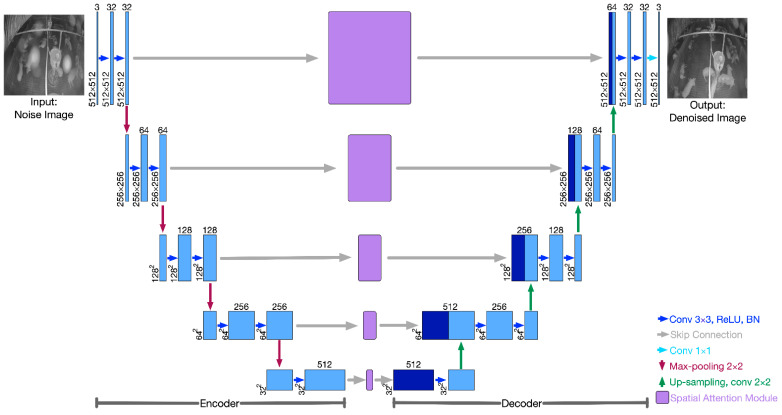
The Generator of the proposed model which consists of three parts: the encoder part, the attention module part, and the decoder part.

The generator is used to remove haze spots in the input noisy image to acquire a corresponding clean image, and it consists of three parts, including an encoder, a decoder, and an attention module. The architecture is illustrated in Figure 4.

The encoder receives a 512×512×3 noisy image, which is then processed with two groups of 3×3 convolutional layers, a ReLU activation function, and batch normalization (BN) operations to obtain a 512×512×32 feature map. Then, the feature map is down-sampled by 2×2 max-pooling operation and used to extract higher-level features in a deeper layer. The number of feature channels is doubled, and the size of the feature map is halved in every deeper layer.

In each layer of the decoder, it first receives the feature maps enhanced by the attention module from the encoder, and then concatenates them with up-sampled features from the next deepest layer. The concatenated feature is processed by two groups of 3×3 convolution operations, including ReLU activation and batch normalization (BN) operations. Then, the obtained feature map is up-sampled by bilinear up-sampling and convolution operations, where the number of feature map channels are halved, the size of the feature map is doubled, and the obtained feature maps are sent to the following layers. The output of the decoder is a 512×512×32 feature map, followed by a 1×1 convolution operation to obtain the denoised output image.

**Figure 5:**
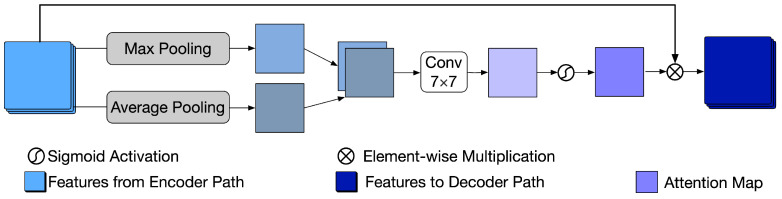
Structure of the proposed spatial attention module.

The attention module receives the feature maps from the encoder in each layer and is trained to obtain several attention maps, which can strengthen the feature transformation in the noisy area while decreasing the feature transformation in the non-noisy area between the noisy image and denoised image at a multi-feature level. It is derived from CBAM [44], where the performance of different types of classical CNN improved significantly when equipped with the attention module. The structure of the attention module is shown in Figure 5. Because applying pooling operations along the channel axis can highlight the informative regions [45], it first applies max-pooling and average-pooling operations to the feature maps from the encoder $F^{encoder}\in R^{H\times W\times C}$ along the channel axis. Then, max-pooling features $F_{mp}^{s}\in R^{H\times W\times 1}$ and the average pooling features $F_{ap}^{s}\in R^{H\times W\times 1}$ are concatenated and processed by a 7×7 convolutional layer followed by a sigmoid function to calculate the attention map $M^{s}\in R^{H\times W\times 1}$. Finally, the features from the encoder are multiplied element-wise with the attention map, and the obtained features are sent to the decoder $F^{decoder}\in R^{H\times W\times C}$. In short, the feature attention process can be calculated as

(2) $$\begin{array}{l} F^{decoder}=F^{encoder}⊗\;M^{s}\left(F^{encoder}\right)\\ =F^{encoder}⊗\;\sigma \left(f^{7\times 7}\left(\left[MaxPool\;\left(F^{encoder}\right);AvgPool\;\left(F^{encoder}\right)\right]\right)\right)\\ =F^{encoder}⊗\;\sigma \left(f^{7\times 7}\left(\left[F_{mp}^{s};F_{ap}^{s}\right]\right)\right) \end{array}$$

where $f^{7\times 7}\left(\cdot \right)$ indicates a 7×7 convolution operation, σ(·) denotes the sigmoid function, and ⊗(·) represents the element-wise multiplication.

Five attention modules are embedded in the U-net, and each receives the features from its previous encoder layer. Instead of defining five different attention modules, we keep these attention modules uniform and ensure that they share the same parameters because the positions and intensities of the haze spots remain the same when downsampling the image features. Thus, the number of additional parameters resulting from adding these five attention modules is 7×7×2=98.

When deployed for use, the generator will receive noisy images collected from the camera and output their corresponding denoised images that would be then used for pig detection. However, during the training process, pairs of noisy and clean images are required for the generator to learn how to correctly transform a noisy image into a clean one. These noisy and clean images used as a pair must represent the same scene with the only difference between them being the noise that appears in the noisy image. Unfortunately, since the images are collected from a pigpen where multiple pigs are constantly moving around, the position of those pigs in the images collected before and after the noise appears is different, making it impossible to obtain pairs of a real-noisy image and a clean image. Thus, as an alternative to real-noisy images, in the training stage, we used simulated noisy images by adding simulated noises to clean images to obtain pairs with consistent scenes. The noise simulation method that was used is introduced and explained in detail in Section 4.1.

#### 3.1.2. Discriminator

**Figure 6:**
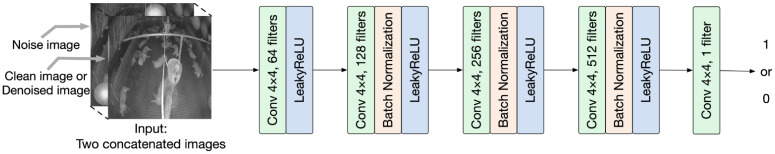
The structure of discriminator.

The discriminator is used to determine whether an image is a clean or denoised image obtained from the generator. It is only used during the training process to force the generator to generate a cleaner image. We adopted the discriminator from the conditional GAN [42,46] in our model because it has the advantage of more stable and faster training by incorporating additional information compared to the original GAN. The input of the discriminator in the original GAN is only the clean image or the denoised image from the generator, whereas the input of the discriminator in the conditional GAN is a concatenation of the noisy image and the clean image or the denoised image from the generator. The architecture of the discriminator is an ordinary CNN model, as shown in Figure 6. When the input is a concatenation of simulated noisy image and clean image, the discriminator is trained to obtain a number that regresses to one. When the input is a concatenation of the simulated noisy image and the image generated by the generator, the discriminator is trained to obtain a number that regresses to zero.

#### 3.1.3. Loss Function

The generator and discriminator are trained alternately during the training process within each iteration. The generator is trained to minimize the following loss function, which consists of four terms, i.e., the adversarial loss $L_{adv}$, the content loss $L_{con}$, the perceptual loss $L_{per}$, and the attention loss $L_{att}$.

(3) $$L=\lambda_{1}L_{adv}+\lambda_{2}L_{con}+\lambda_{3}L_{per}+\lambda_{4}L_{att}$$

where $\lambda_{1},\;\lambda_{2},\;\lambda_{3},\;\lambda_{4}$ are trade-off factors. The generator is trained by minimizing Equation (3).

The adversarial loss $L_{adv}$ is a standard conditional loss function that can increase the stability of the model in the training process.

(4) $$L_{adv}=E_{x}\left[log\left(1-D\left(x,\;G\left(x\right)\right)\right)\right]$$

Here, D denotes the discriminator, G represents the generator, and x refers to the noisy image. The adversarial loss can help the model learn how to generate an image sufficiently similar to the target image in a step-by-step manner.

The content loss is an L1 loss function and can encourage the model to recover the image to the ground truth image as closely as possible.

(5) $$L_{con}=\frac{1}{C\times W\times H}\;\overset{W}{\underset{i=1}{\sum }}\overset{H}{\underset{j=1}{\sum }}\overset{C}{\underset{c=1}{\sum }}{\left|I_{gt}^{i,j,c}-G\left(I_{in}^{i,j,c}\right)\right|}_{1}$$

where $I_{in}$ is the input noisy image, $I_{gt}$ is the ground truth target image, $G\left(I_{in}\right)$ is the image predicted by the generator, and C, W, H represent the number of channels, width, and height of the image, respectively.

Unlike content loss, which compares images in pixel space, perceptual loss [41] compares images in a feature space where the features are extracted from the input images by the VGG19 network. It has been widely applied to several image-to-image translation tasks [27,28,29,47] to maintain perceptual and semantic fidelity.

(6) $$L_{per}=\frac{1}{W_{\alpha ,\beta }\times H_{\alpha ,\beta }}\overset{W}{\underset{x=1}{\sum }}\overset{H}{\underset{y=1}{\sum }}\|\phi_{\alpha ,\beta }{\left(I_{gt}\right)}_{x,y}-\phi_{\alpha ,\beta }{\left(G\left(I_{in}\right)\right)}_{x,y}{\|}_{2}^{2}$$

Here, $\phi_{\alpha ,\beta }\left(\cdot \right)$ represents the feature map obtained by the α-th convolution before the β-th max-pooling layer within an untrainable VGG19 network and $W_{\alpha ,\beta },H_{\alpha ,\beta }$ represent the width and height of the feature map, respectively.

The attention modules in the denoising model calculate several attention maps, which can strengthen the feature transformation in the noisy area while decreasing the feature transformation in the non-noisy area. Instead of producing attention maps without any restrictions, we propose a multi-attention loss to accelerate the regression of the attention modules to the target attention map. The multi-attention loss first sets a target attention map by calculating the element-wise difference between the noisy and clean image, which clearly shows the position and intensity of the noise. The attention module is then trained to produce a predicted attention map that approaches the target attention map. The attention loss can make the attention module trained in a supervised way and ensure the model has a better focus on the noise area. The generator includes five attention modules and adding the attention loss is the sum of the five corresponding attention losses.

(7) $$L_{att}=\overset{5}{\underset{l=1}{\sum }}\|A_{l}-M_{l}{\|}_{2}^{2}$$

Here, A is the predicted attention map generated by the spatial attention module, M is the target attention map calculated by the element-wise difference between the image with the haze spot and the target clean image, and l is the l-th level in the generator. The target attention map M is halved in size at each level in the generator.

After obtaining the generator, the discriminator is trained by maximizing the following loss function.

(8) $$L=E_{x,y}\left[log\left(D\left(x,\;y\right)\right)\right]+E_{x}\left[log\left(1-D\left(x,\;G\left(x\right)\right)\right)\right]$$

Here, D denotes the discriminator, G denotes the generator, x refers to the noisy image, and y is the target clean counterpart.

### 3.2. Pig Detector

After the noisy images are denoised at the preprocessor, the denoised images are sent to the pig detector to perform pig detection. YOLOv5 was selected as the pig detector because it is one of the newest released detectors with the advantages of rapid inference speed, high detection accuracy, and a lightweight network [36,37].

## 4. Experimental Results

The proposed denoising model was derived from the image-to-image translation model and trained to learn pixel-to-pixel mapping from the input noisy image to the target clean image. Hence, the input noisy image and the output clean image should be the same, apart from the noise area that appears in the noisy image and not in the clean image. However, the pigs’ positions in the input noisy image and target clean image are always different owing to their constant movement. Thus, it is impossible to acquire paired data of clean and noisy images to satisfy the training requirements. Synthetics data is a good substitute for real data and has been widely used in previous studies to train diverse models [48,49]. Therefore, we added simulated noise to the clean image to generate paired clean and noisy data to train the model as mentioned at Section 3.1.1. This section introduces the noise simulation method and presents evaluation results for both images with simulated noise and images with real noise. YOLOv5 [37] was used as the pig detector to demonstrate the effectiveness of the denoising model on real-noise data. A comparative experiment of the denoising model with and without the attention module was performed to demonstrate the functionality of the attention module. To demonstrate the effectiveness of the proposed method, we also compared our model with three other state-of-the-art methods, including GridDehaze-Net [31], SpA-GAN for cloud removal [32], and FFA-Net [33], with the source code provided by the original authors.

### 4.1. Data Collection and Datasets

The data were collected using an infrared dome camera (QND-6012R, Hanwha Techwin, Changwon, Korea) located at the inclined top of a pig pen in Gyeongsangnam-do, Korea. Clean and noisy images were collected by the same camera. The clean images (see Figure 1a) were collected on 12 January 2021, the low-level noisy image, which had less noise (see Figure 1c), was collected on 20 January 2021, and the high-level noisy image, which had more noise (see Figure 1d), was collected on 16 February 2021. The collected images had a resolution of 1920×1080 at 10 frames per second (fps) and were resized to 512×512 in the data preprocessor part of the proposed system.

**Figure 7:**
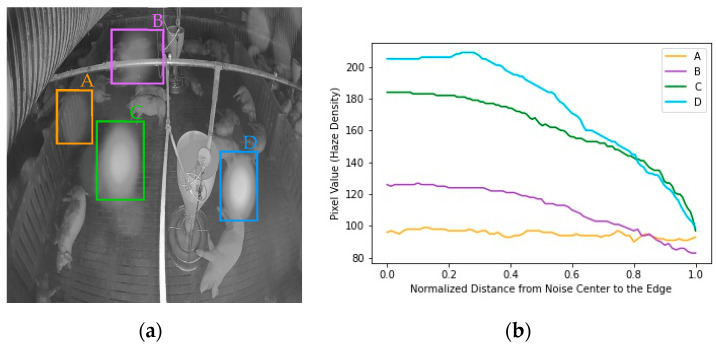
Noise level and variations: (**a**) Four noises appear on the pigpen; (**b**) Noise level and variation from the center to edge of each noise.

The proposed model was trained using paired clean and simulated noisy images. The simulated noise should be as similar as possible to the real noise to maintain the performance of the denoising model. According to the noise simulation method in the image dehazing task, brightness can reflect haze density, whereas the larger the haze density, the larger the brightness [50,51]. The brightness is reflected by the pixel value in the grayscale image [52]. Thus, we analyzed the pixel value of the haze spot in the real-noise image to know the haze spot density changes in the real-noise image. There are four sources of noise in Figure 7a: noise A, noise B, noise C, and noise D. Figure 7b shows that the noise is diverse in terms of intensity and rate of change. Noise A remained uniform from the center to the edge of the noise, noise B and noise C decreased non-linearly from the center to the edge, and noise D remained uniform from the center to a point and then decreased almost linearly to the edge. Owing to the complex nature and variety of real noise, simulating the noise with a monotonous decay causes the trained model to lack adaptation and to exhibit poor robustness to unseen noise. Thus, we used three types of simulated noise, including, uniform noise, liner-form noise, and exponential-form noise, by changing the transmission rate t and the global atmospheric light A in the atmospheric scattering model (Equation (1)) to simulate real noise.

The uniform noise remains consistent; therefore, the transmission rate t and global atmosphere light A are random fixed numbers, where t∈[0.1, 0.4] and A∈[0.6, 1]. The larger of t, the more clearly the pigs may be observed through the noise, and the larger the value of A, the whiter the noise will be.

The linear-form noise varies linearly from the center of the noise to the edge by the following functions:

(9) $$t=d\times T_{edge}+\left(1-d\right)\times T_{center}\;A=\{\begin{array}{cc} A_{center}, & d\times A_{edge}+\left(1-d\right)\times A_{center}>random(0.75,0.85)\\ d\times A_{edge}+\left(1-d\right)\times A_{center} & otherwise \end{array}$$

Here, d∈[0,1] is the normalized distance from a point in the noise to the noise center, $T_{center}=0.1,\;T_{edge}=0.4$ is the transmission rate at the central point and edge of the noise, and $A_{center}=1,\;A_{edge}=0.6$ is the global atmospheric light at the central point and the edge of the noise. We set the atmospheric light that is larger than a random number from 0.75 to 0.85, equal to the atmospheric light at the central position, which is 1, because the real noise remains uniform near the central position, such as noise B in Figure 7a. Finally, Gaussian blur with a filter of 5×5 was used to blur the atmospheric light metrics.

The exponential-form noise varies exponentially from the center of the circle to the edge by the following functions:

(10) $$\begin{array}{l} t=e^{\min\left\{\left(d-0.5\right)\times 4,\;0\right\}}\\ A=e^{-\theta d} \end{array}$$

Here, d∈[0,1] is the normalized distance from a point in the noise to the noise center and θ∈(0.3, 0.7) is a random factor that controls the descent speed of atmospheric light A.

**Figure 8:**
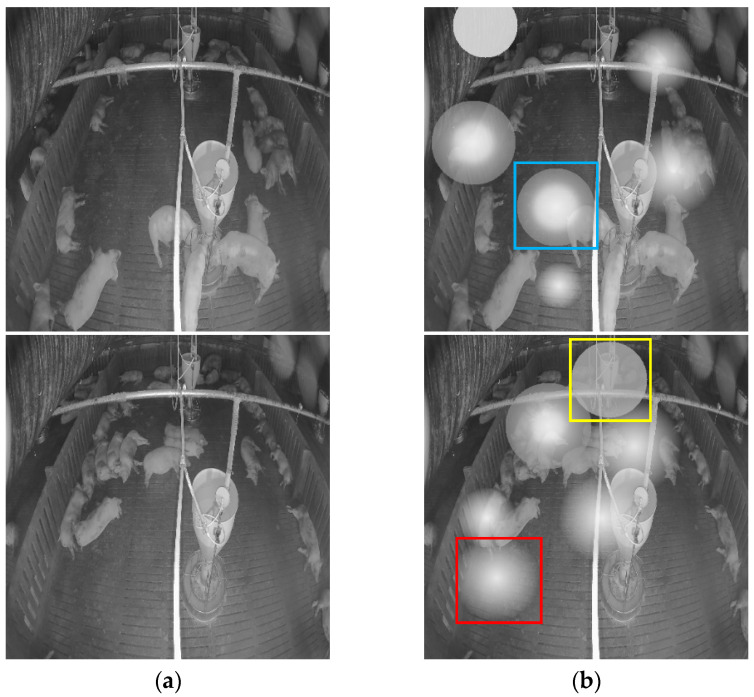
The clean image and simulated noisy image used to train the model. (**a**) Clean image; (**b**) Corresponding noisy image with simulated noise. The uniform noise, linear-form noise, and exponential-form noise are shown in yellow, blue, and red boxes, respectively.

We randomly added roughly from three to eight simulated noise sources to the clean images. The added noise had a 20% chance of being a uniform noise, a 40% chance of being a linear-form noise, and a 40% chance of being an exponential-form noise. In addition, the position of each noise source was random. Figure 8 shows some clean images and the corresponding simulated noisy images. The uniform noise, linear-form noise, and exponential-form noise are shown in yellow, blue, and red boxes, respectively. In total, 18,000 image pairs of clean images and simulated-noisy images were created, with 14,400 image pairs used to train the model and 3600 image pairs used to for testing.

The simulated noisy image is only used for training the model, and the final goal is to remove the haze spots that occurred when using the infrared camera for pig detection in actual pig farms. Thus, YOLOv5 was used to detect pigs in clean images and real-noise images to demonstrate the efficacy of the proposed model in denoising real-noise images. A total of 884 clean images were used for training the detection model, and 359 low-level noisy images and 354 high-level noisy images were used for testing. To reduce the impact of the uninterested area on the detection result, we cropped the pig pen from the original images in both the training and test images in the pig detection experiment.

### 4.2. Experimental Environment and Setup

Both haze spot removal and pig detection experiments were conducted with Python 3.8, PyTorch deep learning library and in an environment with an Intel Core i7-6700K processor with a clock speed of 4.0GHz, 32 GB RAM, an NVIDIA GeForce RTX 3080 GPU, and the Ubuntu 20.04 operation system.

In the image denoising experiment, the trade-off factors $\left\{\lambda_{1},\;\lambda_{2},\;\lambda_{3},\;\lambda_{4}\right\}$ in the loss function were set to {1, 100, 1000, 1}, respectively. {α,β} in the perceptual loss was set to {5,4} to extract the features using the untrained VGG19 network. The image size of the input and output image of the generator is 512×512×3 and the image size of the input image of the discriminator is 512×512×6. Normal initialization was applied to initialize the network. The model was trained for 200 epochs with a batch size of five. The learning rate in the front 100 epochs was fixed at 0.0002 and in the posterior 100 epoch decayed linearly from 0.0002 to 0, gradually. An Adm optimizer with a beta1 value of 0.5 and a beta2 value of 0.999 was used to perform gradient descent.

In the pig detection experiment, YOLOv5-l was selected as the detector for it has the best trade-off between the accuracy and speed among the YOLOv5 family. The image size used in training and testing the detection model is set to 512×512×3. The detection model was trained for 200 epochs with a batch size of 16. The learning rate was set to 0.001 with a decay rate of 0.005. An Adam optimizer with a beta1 value of 0.937 and beta2 value of 0.999 was used to perform gradient descent.

### 4.3. Comparison of Results Obtained with and without the Attention Module

To emphasize the importance of the attention module in our proposed model, we compared the image denoising model with and without the attention module on both the simulated noisy image and the real-noisy image. We used the peak signal-to-noise ratio (PSNR), which is widely used as an image quality evaluation metric, to evaluate the denoising performance of the model with simulated noise. The calculation formula for the PSNR is

(11) $$\mathrm{PSNR}=10⊗\;log_{10}\left(\frac{MAX_{I}^{2}}{MSE}\right)$$

Here, $MAX_{I}$ is the maximum possible pixel value of image I, which is 255 for the 8-bit image and MSE is the mean square error between images I and K, calculated as

(12) $$\mathrm{MSE}=\frac{1}{W\times H}\overset{W}{\underset{i=1}{\sum }}\overset{H}{\underset{j=1}{\sum }}{\left(I\left(i,\;j\right)-K\left(i,\;j\right)\right)}^{2}$$

A larger PSNR value indicates that the predicted image is more similar to the ground truth image and better denoising performance on the simulated noisy image.

We evaluate the denoising performance of the proposed model on real noise by comparing the detection rate of the noisy and denoised images. Precision, recall, and average precision (AP), which are widely used as metrics for evaluating object detection models, were used to evaluate the proposed model and were calculated using the following equations:

(13) $$\begin{array}{l} \mathrm{Precision}=\frac{\mathrm{TP}}{\mathrm{TP}+\mathrm{FP}}\\ \mathrm{Recall}=\frac{\mathrm{TP}}{\mathrm{TP}+\mathrm{FN}}\\ \mathrm{AP}=\overset{1}{\underset{0}{\int }}p\left(r\right)dr \end{array}$$

Here, true positive (TP) case represents the ratio of the overlapping area between the predicted bounding box and ground truth to the summation area of more than 0.5, the false positive (FP) case represents a ratio lower than 0.5, and the false negative (FN) case represents a pig present in the images that the model failed to detect. p(r) represents the precision-recall curve, and AP computes the average value of p(r) over the interval from r=0 to r=1.

Table 2 compares the denoising performance of the proposed model with and without the attention module on the average PSNR and inference time for 3600 synthesized test noisy images. After adding the attention module, PSNR increased from 29.1 dB to 33.3 dB. Although there was a slight increase in the inference time, the performance of the inference time still satisfied the requirements of real-time usage.

**Figure 9:**
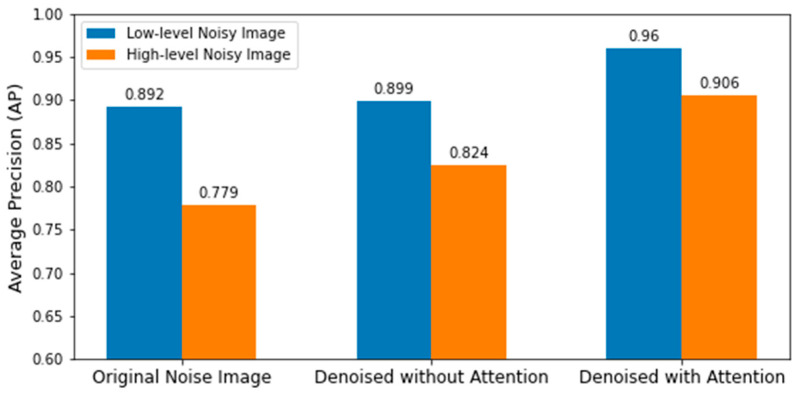
Comparison of average precisions on different level real-noise images and the corresponding denoised images using the denoising model with and without attention module.

Table 3 summarizes the precision, recall, and average precision of low-level real-noise images and the corresponding denoised images of the proposed model with and without the attention module. Table 4 summarizes the precision, recall, and average precision of high-level real-noise images and the corresponding denoised images, with and without the attention module. A graphical representation in Figure 9 shows the comparison of average precisions of low- and high-level real-noise images and the corresponding denoised images, with and without the attention module. All metrics were significantly improved by the model with the attention module, which demonstrates the efficacy of the proposed approach.

**Figure 10:**
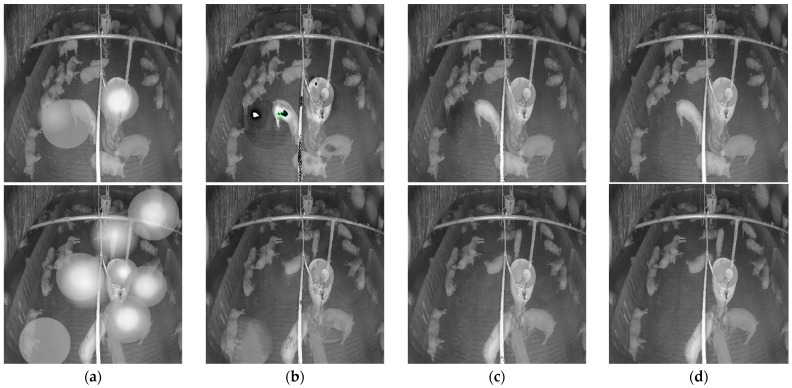
Denoised image produced by the model with and without the attention module on the simulated noisy image: (**a**) simulated noisy image; (**b**) denoised image without attention module; (**c**) denoised image with attention module; (**d**) Clean image.

**Figure 11:**
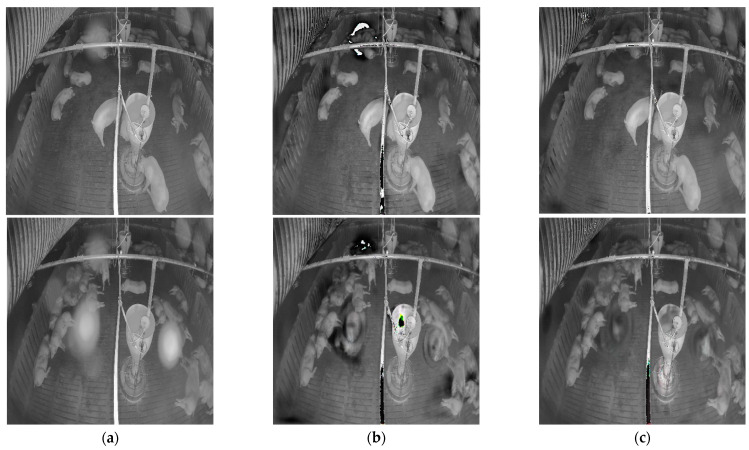
Denoised image produced by the model with and without the attention model on real- noise image: (**a**) real-noise image; (**b**) denoised image without attention module; (**c**) denoised image with attention module.

**Figure 12:**
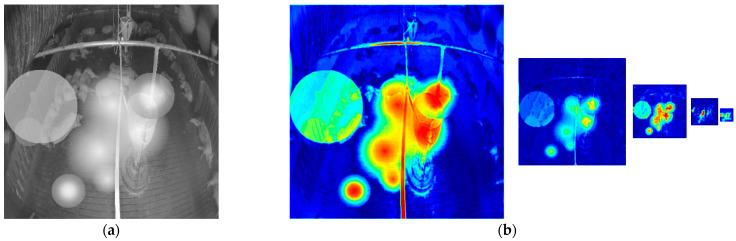
Attention map calculated by the attention module on simulated noisy images: (**a**) simulated noisy image; (**b**) heatmap of the corresponding attention map of each layer in the model.

**Figure 13:**
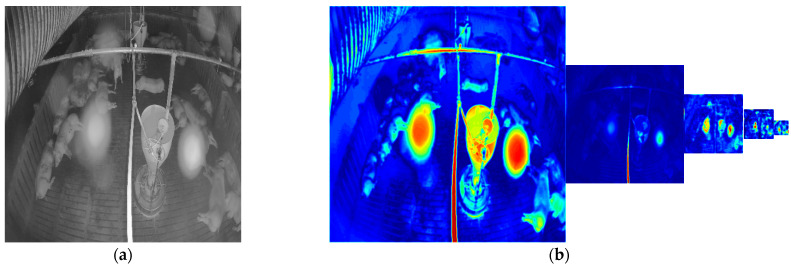
Attention map calculated by the attention module on real-noise images: (**a**) real-noise image; (**b**) heatmap of the corresponding attention map of each layer in the model.

Figure 10 shows the denoised images of the model with and without the attention module on the simulated noisy image. Figure 11 shows the denoised image of the model with and without the attention module on the low-level and high-level real-noise images. Figure 12 and Figure 13 show the attention map calculated by the attention module on the simulated noisy image and high-level real-noise image, respectively. The attention map confirms the attention module can master the position and degree of the noise in a multi-scale very well.

### 4.4. Results of Comparisons with Other Models

We compared our proposed model with three other state-of-the-art methods, including GridDehaze-Net [31], SpA-GAN for cloud removal [32], and FFA-Net [33], with the source code provided by the authors. FFA-Net and GridDehaze-Net were originally designed to perform image dehazing, whereas the haze in their training image appeared continuously at all positions in the image. These two models both crop a smaller image with a size of 240×240 from the center of the input image. In our case, the haze spot is randomly distributed in the image; thus, cropping the image from the center is unadvisable. Thus, the image size in the training and testing datasets of GridDehaze-Net was set to 512×512, whereas in FFA-Net, the training image size was resized to 256×256 because the storage of the GPU could not afford even a single batch of the training of that model. The default image size in the SpA-GAN, which was initially designed for cloud removal and can also be viewed as an image dehazing task, was 512×512, the same as our model, and so we did not modify it.

Table 5 shows a comparison of the denoising performances of each model on the simulated noisy image. As shown in the table, GridDehaze-Net achieved the best PSNR in the simulated noisy image, and our proposed model achieved the fastest inference speed.

**Figure 14:**
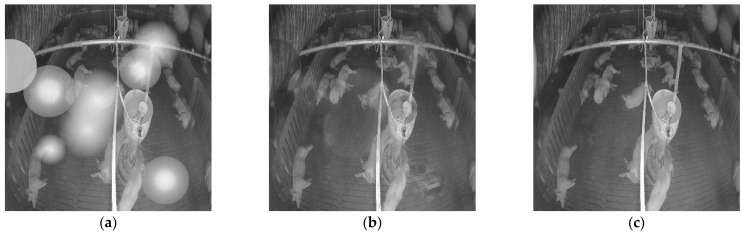

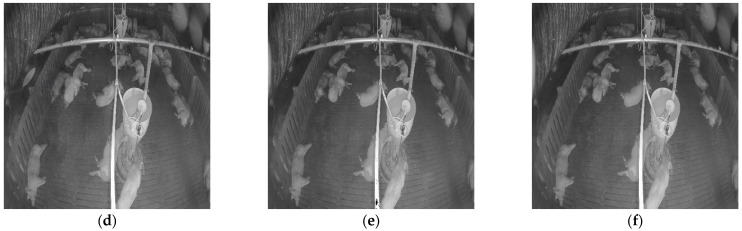
Denoised images on the simulated noisy image by each model: (**a**) simulated noisy image; (**b**) denoised image by FFA-Net; (**c**) denoised image by GridDehaze-Net; (**d**) denoised image by SpA-GAN; (**e**) denoised image by proposed model; (**f**) clean image.

Figure 14 shows the denoised image of the simulated noise for each model. The simulated noisy images were only used for training the model, and the final goal is to remove the noise in the real-noise image and improve the robustness of pig detection in real-noise environments. Thus, the denoising performance of the models for real noise is more critical.

**Figure 15:**
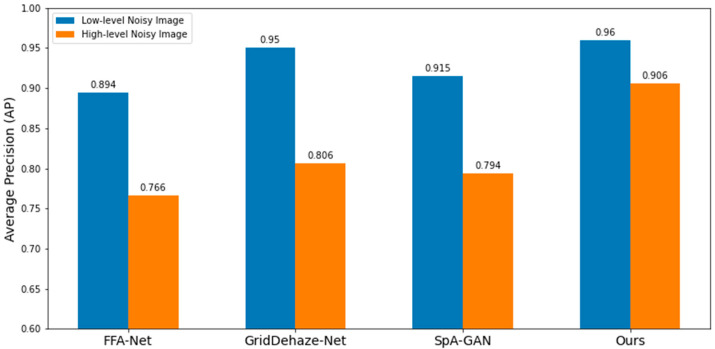
Comparisons of each model’s denoising performance on different level real-noise images.

**Figure 16:**
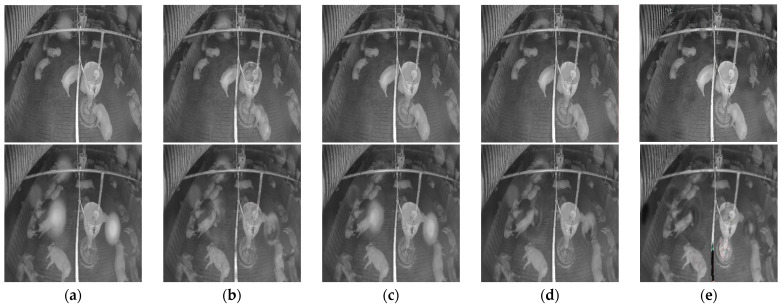
Denoised results on real-noise image by each model: (**a**) Real-noise image; (**b**) Denoised image by FFA-Net; (**c**) Denoised image by GridDehaze-Net; (**d**) Denoised image by SpA-GAN; (**e**) Denoised image by proposed model.

Table 6 compares each model’s denoising performance on the low-level, real-noise image, and Table 7 compares each model’s denoising performance on the high-level, real-noise image. A graphical representation in Figure 15 shows the comparison of each model’s denoising performance on different level real-noise images. The proposed model surpassed the performance of other models on the real-noise images, which confirms the efficacy of the proposed model. Figure 16 shows some denoised results for the low-level and high-level real-noise images.

## 5. Discussion

**Figure 17:**
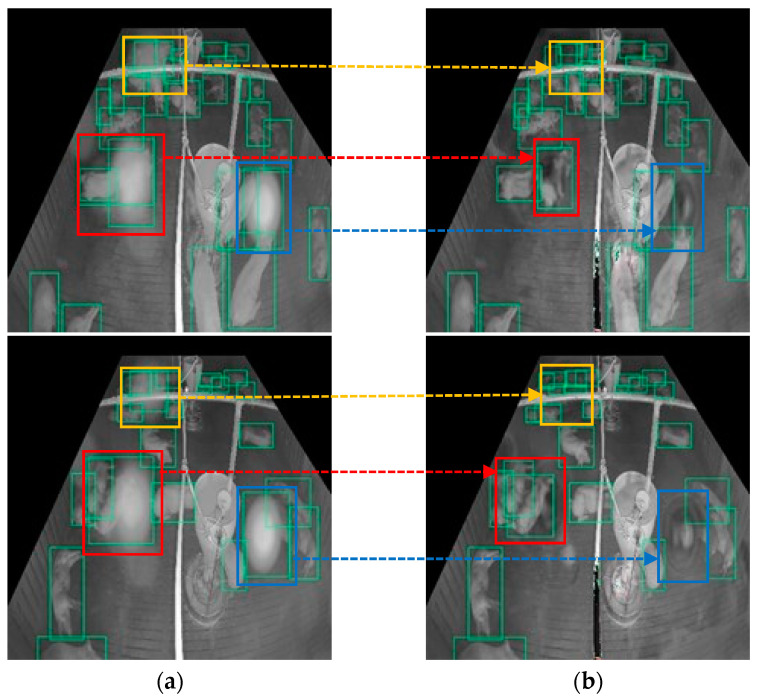
Detection errors in the noisy image are fixed after applying the proposed denoising method: (**a**) pig detection results on real-noisy image, the detection result in the noisy area is highlighted in yellow, red and blue box. (**b**) pig detection results on the denoised image by the proposed model, the detection result on the denoised area in highlighted in yellow, red and blue box.

The results show that our proposed model outperformed those of previous works on images with real noise. This is because the proposed model can first master and locate the degree and position of the noise and then strengthen the image transformation in the noise area at a multiscale level by embedding several attention modules into the U-Net, whereas the previous models consider each area in the image equally or as a single-scale attention map, which does not pay attention to the noise area well, such as the SpA-GAN does. Figure 17 shows the result of solving the failed detection case on the real-noisy image by adding our preprocessing method. In a real-noisy image, the noise was always detected as pigs (false positive), and the pigs covered by the noise could not be detected (false negative). After applying the proposed denoising method, false-positive and false-negative cases were solved.

**Figure 18:**
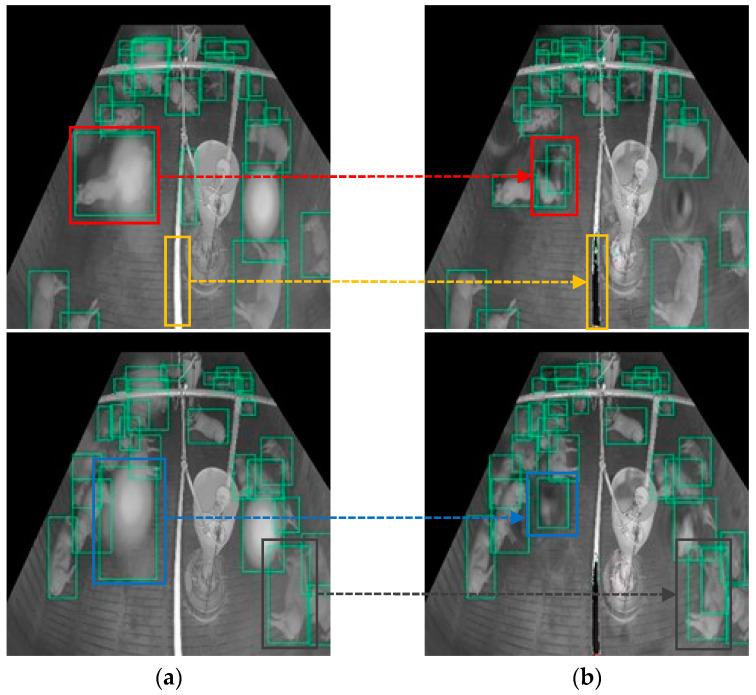
Limitations of the proposed method: (**a**) pig detection results on real-noisy image; (**b**) pig detection results on the denoised image by the proposed model.

Although many errors were solved using our proposed method, some errors did remain. Figure 18 shows some of the limitations of the proposed method. The green boxes represent the bounding boxes of the pigs detected. In the case of the red box, only one pig was covered by the haze spot in the real-noisy image. However, it was detected in two pigs after denoising. In the case of the dark gray box, there was only one pig, and it was detected well in the noisy image, but it was detected as two pigs in the denoised image due to color degradation. In the case of the blue box, the noise area detected as a pig incorrectly remained in the denoised image, although the noise area was reduced. In addition, the obstacle in the yellow box was too close to the camera and caused the IR reflection problem. The proposed model classified it as noise and changed its color from white to black. More specifically, in some cases, the proposed denoising model may distort the non-noise region and it is unable to remove the noise completely. To overcome these limitations, we will consider improving the noise simulation method in future work to improve performance, and we will explore other image denoising methods that do not require the paired noisy image and clean image, so that the model can be trained on unpaired real-noisy images and clean images without noise simulation.

## 6. Conclusions

Automatic pig monitoring is very helpful in managing pigs’ health and welfare problems in intensive pig farms. Among the recently proposed automatic pig monitoring systems, the infrared camera-based method is one of the most popular because it can provide non-invasive and continuous 24 h monitoring. However, when put into practice on actual pig farms, impurities such as the secretion of insects are always deposited on the camera lens, owing to the harsh environment of the farm. This inevitably causes an IR reflection problem, which can seriously affect the performance of pig automatic monitoring systems.

In this study, we have proposed a noise-robust, real-time pig detection system that can improve pig detection accuracy in actual farms where infrared cameras suffer from IR reflection problems. The system consists of a data collector to collect infrared images, a preprocessor to transform noisy image into clean images, and a detector to detect pigs. The preprocessor embedded a multi-scale spatial attention module, which enables the model to pay more attention to the noise area into U-net and GAN models. A multi-scale attention loss has been proposed to accelerate the regression of the attention model. A noise simulation method was also proposed to enable the model to be trained on paired noisy and clean data. The model can operate in in a real-time and end-to-end manner.

The experimental results have shown that the proposed method was able to significantly improve the average precision of detection from 0.766 to 0.906 when the pig detection system was affected by the IR reflection problem. Compared with recent state-of-the-art image dehazing methods, the proposed method was able to remove this special type of noise better, and its execution speed was faster. Although the proposed method outperformed existing methods in dealing with real noise, considerable room for improvement remains. In future research, we intend to collect more diverse data to reverify the model performance on different scenarios with different camera locations and improve the denoising performance by improving the noise simulation method. Also, we will explore other image denoising methods that do not require the paired noisy image and clean image, so that the model can be trained on unpaired real-noisy image and clean image without noise simulation.

## Figures and Tables

**Table 1:** Some recent studies on image dehazing (Published between 2011–2021).

Haze Type	Haze Intensity	Haze Position	Image Type	End-to-End Point	Dehazing Technique	Real Time	References
Successive	Related to scene depth	The whole image	RGB	No	Prior-based	No	[23]
				No	Prior-based	No	[24]
				No	CNN	No	[26]
				No	CNN	No	[27]
				Yes	GAN	Not Specific	[28]
				Yes	GAN	Yes	[29]
				Yes	U-net	No	[30]
				Yes	Custom network + Attention	Not Specific	[31]
				Yes	Custom network + Attention	Not Specific	[33]
Scattered	Random	Random	Grayscale	Yes	GAN + U-net + Attention	Yes	Proposed

**Table 2:** Comparisons of the proposed model with and without the attention module on the average PSNR and inference time.

	Denoised Image without Attention Module	Denoised Image with Attention Module
PSNR (dB)	29.1	33.3
Inference time (ms)	4.06	4.81

**Table 3:** Precision, recall, and average precision (AP) on low-level real-noise images and the corresponding denoised images with and without attention module.

	Low-Level Real-Noise Image	Denoised Image without Attention Module	Denoised Image with Attention Module
Precision	0.890	0.892	0.936
Recall	0.823	0.839	0.927
AP	0.892	0.899	0.960

**Table 4:** Precision, recall, and average precision (AP) on high-level noisy images and the corresponding denoised images with and without attention module.

	High-Level Real-Noise Image	Denoised Image without Attention Module	Denoised Image with Attention Module
Precision	0.819	0.839	0.879
Recall	0.764	0.779	0.853
AP	0.779	0.824	0.906

**Table 5:** Comparison of the denoising performance of each model on simulated noisy images.

	FFA-Net	GridDehaze-Net	SpA-GAN	Proposed
PSNR (dB)	28.6	**43.3**	31.9	33.3
Inference time (ms)	78.0	14.7	10.8	**4.8**

**Table 6:** Comparison of each model on the low-level noisy image.

	FFA-Net	GridDehaze-Net	SpA-GAN	Proposed
Precision	0.885	0.922	0.882	**0.936**
Recall	0.825	0.901	0.866	**0.927**
AP	0.894	0.950	0.915	**0.960**

**Table 7:** Comparison of each model on the high-level noisy image.

	FFA-Net	GridDehaze-Net	SpA-GAN	Proposed
Precision	0.773	0.783	0.785	**0.879**
Recall	0.717	0.833	0.756	**0.853**
AP	0.766	0.806	0.794	**0.906**

## Data Availability

Not applicable.
